# Adherence to Evidence‐Based Guidelines and Implications When Designing Electronic Documentation for Urinary Catheters

**DOI:** 10.1111/jocn.17459

**Published:** 2024-10-06

**Authors:** Bothe Janine, Lagat Sheena, Rebecca Crellin, Kelly‐Ann Hahn, Patton Vicki

**Affiliations:** ^1^ Department of Surgery St George Hospital Sydney Kogarah New South Wales Australia; ^2^ Curtin University School of Nursing Bentley Western Australia Australia; ^3^ Department of Women's Health Royal Prince Alfred Hospital Camperdown New South Wales Australia; ^4^ Nursing Quality & Safety and Acute Care Research Unit Royal Perth Bentley Group Perth Western Australia Australia

**Keywords:** CAUTI, documentation, indwelling urinary catheters, management, practice

## Abstract

**Aim:**

The aim of this study was to investigate the point prevalence and the rate of adherence to evidence‐based guidelines for patients who had indwelling urinary catheters in three Australian acute care hospitals.

**Design:**

A cross‐sectional observational design was used.

**Methods:**

A multisite cross‐sectional observational design was utilised in three acute hospitals across Australia. Data were collected from each site in a single day directly from observation of the patient, the bedside notes and medical records. The data collected included observations of clinical care and scrutiny of the documentation of the insertion details and catheter care using best practice guidelines.

**Results:**

Of the 1730 patients audited, 47% were female. The mean point prevalence of catheters in situ across three sites was 12.9%. Correct documentation compliance was reported to be, on average, 40%. Documentation was significantly better when a template was available to guide information recorded: this was regardless of whether it was hard copy or electronic. Overall, clinical care compliance with best practices was 77%. Of note for improvement was the fixing of the urinary catheter to the thigh in highly dependent patients.

**Conclusion:**

It was identified that there is a need for improvement across all three sites: specifically regarding securement of the urinary catheter to the patient’s thigh within the ICU. In addition, it was identified that there is a need for documentation of the urine bag change in ward areas. Documentation may be improved by incorporating templates into healthcare documentation systems in the future. Further work is needed to ensure nurses are aware of the adverse effects of urinary catheters and thus, the need to adhere to best practice guidelines.

**Patient or Public Contribution:**

There has been no patient or public contribution.

**Reporting Method:**

We have adhered to the STROBE guidelines for reporting.

**Summary**

- What problem did the study address?
  ○ The study examined the rates of adherence to documentation and clinical care to best practice guidelines in patients with a urinary catheter.
- What were the main findings?
  ○ Documentation compliance is poor but significantly better when a template is used regardless of whether it is electronic or hard copy.
  ○ Education of nurses to highlight the importance of securing a catheter to the patient’s thigh is required.
- Where and on whom will the research have an impact?
  ○ The primary impact of the research is on nurses caring for patients with an indwelling catheter (IDC) in an acute care hospital.
  ○ Those designing software for patient clinical record documentation in acute care should consider incorporating a template for IDC insertion to improve documentation compliance.
  ○ Nurses caring for patients with urinary catheters in an acute setting need to be vigilant in the technique of insertion, management and the timely removal of this invasive device which poses a significant risk to the patient.
  ○ Nurses are required to demonstrate through their documentation that they have provided evidence‐based care. This paper has identified how nurses can improve this documentation by utilising a template.
  ○ This research has provided a benchmark that other acute care hospitals may use to measure against and improve care of the patients with an IDC.
- What does this paper contribute to the wider global clinical community?
  ○ Identifies the current IDC prevalence average in three acute care hospitals as 12.9%.
  ○ It has been identified that nurses do not treat an indwelling urinary catheter as an invasive medical device as they rarely document device batch numbers.
  ○ A documentation template, either electronic or hard copy, yields more thorough documentation of IDC insertion and should be considered when designing future software.
  ○ Identifying areas of improvement in IDC care and documentation may contribute to reduction in catheter‐associated urinary tract infection by timely removal of IDC, appropriate securement and reduction in contamination. These improvements may avoid costly complications, expedite patient restoration of health and reduce overall healthcare costs.

## 1 Introduction

A healthcare‐acquired infection (HAI) is an infection which was not present at the time of admission but acquired during the person's episode of care in a healthcare setting (Shamshiri et al. 2016). In Australia, approximately 60,037 hospital‐acquired infections (HAI) occurred in 2015–2016; an estimated one in every 74 hospitalisations. Of these, 26.6% were attributed to catheter‐associated urinary tract infections (CAUTIs) (Australian Commission on Safety and Quality in Health Care 2018). A CAUTI is defined as an infection occurring in those whose urinary bladder is catheterised or has been catheterised within the past 48 h (Foxman 2010).

On average, patients with a hospital‐acquired urinary tract infection (UTI) have an extended hospital stay of 20.6 days which incurs an additional AU$42,724 in healthcare costs (Australian Commission on Safety and Quality in Health Care 2018). These HAIs significantly increase the risk of mortality and morbidity for patients and the risk is even higher among the elderly, female, diabetic cohorts and/or patients who have prolonged urinary catheterisation (Cromwell and Crespo‐Diaz 2014; Australian Commission on Safety and Quality in Health Care 2018; Werneburg 2022).

The insertion, management and removal of urinary catheters are primarily the responsibility of nurses (Jones et al. 2022). If each of these tasks does not adhere to best practice standards, the development of a CAUTI may occur (Cromwell and Crespo‐Diaz 2014). Evidence‐based clinical guidelines include catheter management which encompasses preinsertion, postinsertion and ongoing management until the removal of the urinary catheter (Werneburg 2022).

In addition, and of significant importance, is the removal of indwelling catheters (IDCs) as soon as it is clinically indicated (Flores‐Mireles, Hreha, and Hunstad 2019; Giles et al. 2019; Gustafsson et al. 2019; Agency for Healthcare Research and Quality October 2015). There is an increased 3%–7% risk of developing a UTI every day that the catheter remains in situ (Werneburg 2022). This risk arises because the catheter damages the mucosal wall and exposes the cells to bacteria introduced as the patient's own native microflora at the time of insertion either intraluminal or extraluminally (Leranoz et al. 1997; Warren 2001).

To provide optimal clinical care, it is essential that there is accurate documentation around the catheter insertion, management and removal for clinicians to refer to (Okeke et al. 2023).

### 1.1 Background

Monitoring an organisation's incidence and management of urinary catheters including the subsequent clinical documentation is an important component of CAUTI prevention. This has been highlighted in the Australian Commission on Safety and Quality in Health Care (Australian Commission on Safety and Quality in Health Care 2018) best practice document with suggested improvements to decrease the rates of hospital‐acquired infections, including those related to urinary tract infections and more specifically CAUTIs.

Examination of the published literature identified only 12 studies focussing on indwelling urinary catheters, compliance with evidence‐based guidelines and accurate medical record documentation (Adib‐Hajbaghery and Aghajani 2009; Cromwell and Crespo‐Diaz 2014; Andreessen, Wilde, and Herendeen 2012; Fink et al. 2012; d'Stere Murphy et al. 2018; Shamshiri et al. 2016; So et al. 2014; Wynne et al. 2013; Rhodes et al. 2014; Appah, Hunter, and Moore 2016; Parker et al. 2017; Coventry et al. 2021). Collectively these studies report a narrow range of point prevalence of urinary catheters in situ centring around 12% within acute care organisations (Giles et al. 2019; Coventry et al. 2021). Four of these studies were from Australian centres (Giles et al. 2019; Coventry et al. 2021; Rhodes et al. 2014; Parker et al. 2017).

The majority of the 12 studies reported data collection within an acute care setting and demonstrated inadequate compliance rates with documentation in all aspects of urinary catheter insertion and daily care. In contrast, the clinical observations reported higher rates of compliance around the clinical care such as catheter cleanliness, draining freely and collection bags kept below the level of the bladder. The area of clinical care that was identified as the greatest need for improvement was the securement of the catheter. The compliance for this parameter was between 17% and 62% (Adib‐Hajbaghery and Aghajani 2009; Coventry et al. 2021; d'Stere Murphy et al. 2018; Rhodes et al. 2014; Shamshiri et al. 2016; Wynne et al. 2013).

Despite the high utilisation of urinary catheters in an acute care setting and the potential for complications, there are relatively few studies examining compliance to evidence‐based practice and the associated documentation reporting the nursing care administered.

### 1.2 Aim

The aim of this study was to investigate the point prevalence and the rate of adherence to evidence‐based guidelines in three Australian hospitals for inpatients who had IDC in situ.

## 2 Methodology

### 2.1 Study Design

This study utilised a cross‐sectional observational design to report on the clinical care and documentation compliance measured against best practice tool identified by Coventry et al. (2021). The study also calculated the point prevalence of urinary catheter usage.

### 2.2 Study Settings

This study was undertaken in three hospitals across Australia. Hospital A is a New South Wales (NSW) metropolitan‐accredited, principal teaching referral hospital of 624 beds. Hospital B is a NSW metropolitan tertiary and quaternary referral hospital with 1200 beds including a large mental health unit. Hospital C is in Western Australia (WA) and is an acute tertiary hospital of 638 beds with a further subacute site of 199 beds.

### 2.3 Inclusion Exclusion Criteria

All inpatients with an IDC in situ at the time of data collection in the three organisations were considered for the study. Exclusion criteria of this group of patients included any outpatients in the organisation, those who could not give consent, those who were unavailable and off the ward (e.g., were in radiology or theatre), secure mental health units and those patients under the age of 18. Children's wards were omitted from the study.

### 2.4 Study Activity

All patients with a urinary catheter in situ were identified. The clinical care was examined, and the following observations were noted: IDC secured to thigh, IDC visibly clean, urine draining freely, closed system maintained, drainage below bladder, drainage bag off the floor, drainage bag less than two‐thirds full and date change on drainage bag.

All medical documentation (including eMR, bed charts and notes) records of those patients with a urinary catheter were checked for the indication for IDC, the date IDC was inserted, the name of the person who inserted IDC, designation of person who inserted catheter, catheter size, catheter type, catheter batch number, volume inserted in balloon, volume of urine drained on insertion, alternative option to catheter, planned removal date, fluid balance chart completed, daily perineal hygiene and presence of a CAUTI.

### 2.5 Data Collection and Data Analysis

Each hospital carried out its organisational data collection in a single day. This was completed by multiple pairs of Registered Nurses (RNs) who had undergone education to use the tool. The pairing of nurses collecting the data enhanced a consistent and nonbiased approach. The process commenced with the two nurses informing the Nurse Unit Manager of the IDC audit. Next, the RNs approached the team leader to identify patients with an IDC. These patients were then approached and informed about the study and were provided a handout. Verbal consent was obtained from the patients witnessed by the two RNs and was documented. Data were collected from the patient assessment, from electronic or paper medical records and any bedside charts.

Data were either entered directly into REDCap using tablets at the time of collection or on paper and later entered into REDCap. These data were downloaded into SPSS v29 (IBM) for analysis.

Descriptive statistics was used to analyse the proportion of patients, mean in normally distributed data and median in non‐normally distributed data. Commonalities and differences between and within the hospitals were documented. Point prevalence and audit rate were calculated. Differences between groups were calculated using independent T‐test and ANOVA. Missing data were approached utilising complete case analysis. Data were reported using STROBE reporting guidelines (von Elm et al. 2008).

### 2.6 Ethical Considerations

Low‐risk multisite ethics was obtained in New South Wales: HREC 2020/ETH03054; and single‐site low risk review was received in Western Australia: GEKO 2020/PID03438.

## 3 Results

A total of 1730 patients were audited and there were more males (*n* = 916) than females (*n* = 753) or others (*n* = 61) in those who were audited. Of these 1730 patients, 224 had an IDC which gave an overall mean point prevalence rate of 12.9%. There were 19 patients with IDCs who were not audited as they were unavailable, did not or were unable to give consent or were not present on the ward (e.g., in operating theatres). This gave an overall audit rate of 92%. The breakdown by hospital can be found in Table 1.

**TABLE 1:** Comparative table of total number of patients, number of patients with an IDC and point prevalence.

	Hospital A	Hospital B	Hospital C	Overall
Total number of patients in study	525	451	754	1730
Gender				
Male	56%	36%	61%	*n* = 916 (53%)
Female	44%	52%	38%	*n* = 753 (44%)
Other	0	12%	1%	*n* = 61 (3%)
Total number of those with IDC	62	72	90	224
Point prevalence	11.8%	16%	11.9%	12.9%
Those with IDC not audited	2	11	6	19
Audit rate	97%	85%	93%	92%

### 3.1 Documentation Results

The mean compliance rate with best evidence in IDC insertion documentation was 40%. The overall mean documentation of the batch number of the device at the time of insertion was 2% while the overall mean rate for documentation of the date of insertion was 79% and documentation of a fluid balance chart was 90%. The breakdown for each of the documentation audit points for each hospital can be found in Table 2.

**TABLE 2:** Multiple comparisons of documentation compliance for insertion of IDC between the hospitals.

Dependent variable		Hospital	Location	Mean difference	SE	*p* ^a^	
	Percentage compliance						Test
Date of catheter insertion documented	86%	Hospital A	Hospital B	0.227	0.075	0.009	ANOVA
			Hospital C	0.102	0.067	0.386	ANOVA
	67%	Hospital B	Hospital A	−0.227	0.075	0.009	ANOVA
			Hospital C	−0.125	0.070	0.231	ANOVA
	78%	Hospital C	Hospital A	−0.102	0.067	0.386	ANOVA
			Hospital B	0.125	0.070	0.231	ANOVA
Name of person who inserted the catheter documented	71%	Hospital A	Hospital B	0.498^a^	0.086	< 0.001	ANOVA
			Hospital C	0.055	0.076	1.000	ANOVA
	23%	Hospital B	Hospital A	−0.498^a^	0.086	< 0.001	ANOVA
			Hospital C	−0.443^a^	0.080	< 0.001	ANOVA
	68%	Hospital C	Hospital A	−0.055	0.076	1.000	ANOVA
			Hospital B	0.443^a^	0.080	< 0.001	ANOVA
Designation of person who inserted the catheter documented	61%	Hospital A	Hospital B	0.403^a^	0.090	< 0.001	ANOVA
			Hospital C	0.026	0.080	1.000	ANOVA
	23%	Hospital B	Hospital A	−0.403^a^	0.090	< 0.001	ANOVA
			Hospital C	−0.376^a^	0.083	< 0.001	ANOVA
	61%	Hospital C	Hospital A	−0.026	0.080	1.000	ANOVA
			Hospital B	0.376^a^	0.083	< 0.001	ANOVA
Catheter size documented	68%	Hospital A	Hospital B	0.027	0.088	1.000	ANOVA
			Hospital C	−0.002	0.078	1.000	ANOVA
	67%	Hospital B	Hospital A	−0.027	0.088	1.000	ANOVA
			Hospital C	−0.029	0.082	1.000	ANOVA
	70%	Hospital C	Hospital A	0.002	0.078	1.000	ANOVA
			Hospital B	0.029	0.082	1.000	ANOVA
Catheter type documented	44%	Hospital A	Hospital B	−0.185	0.094	0.156	ANOVA
			Hospital C	−0.050	0.084	1.000	ANOVA
	64%	Hospital B	Hospital A	0.185	0.094	0.156	ANOVA
			Hospital C	0.135	0.088	0.382	ANOVA
	50%	Hospital C	Hospital A	0.050	0.084	1.000	ANOVA
			Hospital B	−0.135	0.088	0.382	ANOVA
Catheter batch number documented	2%	Hospital A	Hospital B	−0.022	0.023	1.000	ANOVA
			Hospital C	0.017	0.021	1.000	ANOVA
	4%	Hospital B	Hospital A	0.022	0.023	1.000	ANOVA
			Hospital C	0.038	0.022	0.232	ANOVA
	0%	Hospital C	Hospital A	−0.017	0.021	1.000	ANOVA
			Hospital B	−0.038	0.022	0.232	ANOVA
Volume inserted into the balloon documented	58%	Hospital A	Hospital B	0.465^a^	0.087	< 0.001	ANOVA
			Hospital C	0.022	0.078	1.000	ANOVA
	14%	Hospital B	Hospital A	−0.465^a^	0.087	< 0.001	ANOVA
			Hospital C	−0.444^a^	0.081	< 0.001	ANOVA
	57%	Hospital C	Hospital A	−0.022	0.078	1.000	ANOVA
			Hospital B	0.444^a^	0.081	< 0.001	ANOVA
Volume of urine drained on insertion documented	39%	Hospital A	Hospital B	0.227	0.084	0.023	ANOVA
			Hospital C	0.138	0.075	0.202	ANOVA
	17%	Hospital B	Hospital A	−0.227	0.084	0.023	ANOVA
			Hospital C	−0.089	0.078	0.775	ANOVA
	26%	Hospital C	Hospital A	−0.138	0.075	0.202	ANOVA
			Hospital B	0.089	0.078	0.775	ANOVA
Planned date for removal of catheter documented	18%	Hospital A	Hospital B	0.105	0.069	0.385	ANOVA
			Hospital C	0.005	0.061	1.000	ANOVA
	8%	Hospital B	Hospital A	−0.105	0.069	0.385	ANOVA
			Hospital C	−0.100	0.064	0.359	ANOVA
	18%	Hospital C	Hospital A	−0.005	0.061	1.000	ANOVA
			Hospital B	0.100	0.064	0.359	ANOVA
Fluid balance chart documented	93%	Hospital A	Hospital B	0.101	0.055	0.195	ANOVA
			Hospital C	0.074	0.049	0.394	ANOVA
	87%	Hospital B	Hospital A	−0.101	0.055	0.195	ANOVA
			Hospital C	−0.027	0.051	1.000	ANOVA
	89%	Hospital C	Hospital A	−0.074	0.049	0.394	ANOVA
			Hospital B	0.027	0.051	1.000	ANOVA
Daily perineal/penile hygiene documented	47%	Hospital A	Hospital B	0.346^a^	0.084	< 0.001	ANOVA
			Hospital C	0.250	0.075	0.003	ANOVA
	15%	Hospital B	Hospital A	−0.346^a^	0.084	< 0.001	ANOVA
			Hospital C	−0.096	0.078	0.652	ANOVA
	25%	Hospital C	Hospital A	−0.250	0.075	0.003	ANOVA
			Hospital B	0.096	0.078	0.652	ANOVA

^a^ Bonferroni—adjusted to the level of 0.003.

**TABLE 3:** Documentation compliance in those with and without a sticker template in the medical record progress note within Hospital C.

Audit point	Sticker in notes	Sticker not in notes	*p*	Chi‐squared test
Date of insertion documented	100%	50%	< 0.01	Fischer's exact 2 sided
Name of person who inserted IDC documented	96%	27%	< 0.01	Fischer's exact 2 sided
Designation of person who inserted IDC documented	79%	32%	< 0.01	Fischer's exact 2 sided
Size of IDC documented	98%	32%	< 0.01	Fischer's exact 2 sided
Catheter type documented	71%	21%	< 0.01	Fischer's exact 2 sided
Volume in balloon documented	85%	18%	< 0.01	Fischer's exact 2 sided
Volume of urine on insertion documented	33%	18%	N/S*	Fischer's exact 2 sided
Planned date for removal documented	29%	3%	0.003	Pearsons chi‐squared
Hygiene documented	14%	18%	N/S*	Fischer's exact 2 sided

* Not significant *p* > 0.05.

The compliance for documentation in those patients with a template for completing the IDC insertion details was significantly higher when compared to those without a template (Tables 2, 3, 4). Of the two hospitals with electronic medical records (eMRs), only one had a template within the software. The non‐eMR hospital had a sticker that was completed within the medical record and affixed to the progress notes.

**TABLE 4:** Difference in documentation in those patients in Hospital A with an electronic template and Hospital B without electronic template within electronic medical record.

Audit point	Documented in Hospital A	Documented in Hospital B	*p*	Chi‐square test performed
Date of insertion documented	54%	35%	0.04	Fischer's exact 2 sided
Name of person who inserted IDC documented	44%	12%	< 0.01	Fischer's exact 2 sided
Designation of person who inserted IDC documented	38%	12%	< 0.01	Fischer's exact 2 sided
Size of IDC documented	42%	35%	N/S*	Fischer's exact 2 sided
Catheter type documented	27%	33%	0.05	Fischer's exact 2 sided
Volume in balloon documented	36%	7%	< 0.01	Fischer's exact 2 sided
Volume of urine on insertion documented	24%	9%	0.012	Fischer's exact 2 sided
Planned date for removal documented	11%	4%	N/S*	Pearson Chi‐square 2 sided
Hygiene documented	29%	8%	< 0.01	Fischer's exact 2 sided

* Not significant: *p* > 0.05.

### 3.2 Observation of Care Results

Overall, the mean percentage of compliance with best evidence in the management of the IDC was 77%. The two lowest compliance points were the date of the last drainage bag change indicated on the bag (15%) and the securing of the IDC to the patient's thigh (58%). The breakdown for each hospital can be found in Table 5. There were only two audit points that were significantly different between the hospitals. These were the securement of catheter and placing the drainage bag off the floor. Hospital C was significantly better at securement (*p* < 0.001) and the poorest at keeping the drainage bag off the floor (*p* < 0.001) as seen in Table 5.

**TABLE 5:** Clinical care audit results.

Clinical care audit point	Hospital A % compliance	Hospital B % compliance	Hospital C % compliance	Overall % compliance	*p*	Chi‐squared test
IDC secured to thigh	40%	42%	78%	58%	< 0.001	Pearson
IDC visibly clean	90%	87%	96%	92%	N/S*	Pearson
Urine draining freely	94%	92%	95%	94%	N/S*	Pearson
Closed system maintained	94%	90%	97%	94%	N/S*	Pearson
Drainage below bladder	90%	92%	93%	92%	N/S*	Pearson
Drainage bag off the floor	89%	89%	77%	84%	< 0.001	Pearson
Drainage bag <2/3 full	86%	85%	93%	88%	N/S*	Pearson
Date change on drainage bag	8%	48%	1%	15%	N/S*	Pearson
Mean % compliance for clinical care	74%	78%	79%	77%		

* Not significant *p* > 0.05.

## 4 Discussion

This study of three Australian hospitals demonstrated a point prevalence rate of IDC ranging from 11.9% to 16% giving an average across the three hospitals in Australia at 12.9%. This overall point prevalence is in keeping with other reported rates in acute settings (Shackley et al. 2017). The lower the point prevalence of IDCs in an organisation, the lower the risk of hospital‐acquired UTIs and thus the associated costs and length of stay (Agency for Healthcare Research and Quality 2015). This is because practices such as avoiding the use of an IDC where possible, or at least the removal of an IDC as soon as possible, lessens the risk of exposure of the patient to introduced bacteria to a damaged bladder mucosal wall during catheterisation: thus a lower point prevalence may reflect a better standard of care (Giles et al. 2019; Gustafsson et al. 2019).

Despite the significant consequences of substandard management of catheters, nurses’ knowledge and attitudes around evidence‐based catheter care remain limited. This is evidenced by the poor compliance with documentation in this study. Nurses need to consider the importance of documentation of the details of insertion, removal and type of catheter inserted to assist in communicating with all members of the healthcare team.

Our data indicated there were very few catheters with a planned removal date documented and this is concerning as the relationship between extended dwelling and increased CAUTI is well documented (Leranoz et al. 1997; Warren 2001; Hernandez, King, and Stewart 2019). To overcome this, nurse‐led (also referred to as criteria‐led) removal of IDCs may reduce the risk of harm (Giles et al. 2019; Jones et al. 2022). Some criteria‐led practices that dictate the day and timing of the IDC removal result in a more successful trial of void with larger volume of urine passed (Kelleher 2002).

Other criteria‐led practices, are documented in clinical pathways, such as for those with an Enhanced Recovery After Surgery (ERAS) programme. This programme, used in one of the studied hospitals, recommends the removal of any urinary catheter on Day 1 postoperative wherever possible (Meillat et al. 2021). However, the use of criteria‐driven practice is not widespread in these organisations and, more commonly, the removal is initiated through discussion with the patient's medical staff.

The insertion of an IDC is generally the decision of the medical team and so a qualitative study investigating the clinical reasoning to insert an IDC during a patient's hospitalisation may be beneficial in understanding why such a decision is reached (Fernández‐Ruiz et al. 2013). The reasoning for the insertion of IDC could not be ascertained in this study due to the poor documentation around both the clinical indication for IDC and the amount of urine drained in the first 10–15 min after catheterisation. The lack of this documentation may have direct adverse clinical consequences (Pandian and Drake 2016), especially in those patients presenting with urinary retention. Accurate documentation has been shown to assist in identifying acute or acute on chronic retention and influencing treatment for better patient outcomes (Emberton and Fitzpatrick 2008).

In general, documentation was poorly completed in all hospitals. This is generally attributed by staff to the challenge of the amount of time it takes to document correctly, and the number of admissions in the ward (Mutshatshi et al. 2018). Hospital B's documentation compliance was particularly low at an overall score of 31%. Poor documentation of clinical care given may be influenced by ‘end of shift’ documentation (Govasli and Solvoll 2020) where it is the ‘final task to complete’ before going home, rather than contemporaneously documenting. Furthermore, if the documentation around IDC insertion and subsequent management is being completed at the end of the shift and not at the time of insertion or care, there may be details forgotten or omitted.

It is evident in this study that the use of a template to document insertion details yielded better completion rates. Hospital A has an electronic template (see Image 1) which contains specific fields for IDC insertion while Hospital C was still utilising paper documentation, with a sticker template placed into the patient's notes (Image 2). Although Hospital B uses eMR, there is no template. This may indicate that as we all move to electronic documentation, the importance of templates to prompt the entry of specific information should be highlighted during software development.

During the audit of clinical care, it was noted that all care provided was far more evidence‐based and consistent across the three centres than the documented care with only two audit points significantly different across the sites. In other reports, some authors reported that the quality of documentation compliance was related to the quality of clinical care (Duclos‐Miller 2016; McCarthy et al. 2019). However, our data do not reflect those findings, as the level of clinical care was generally very good if not excellent, and it is the documentation that requires improvement. This indicates that while nurses and midwives know what best practice is required, the care and management they deliver are not always fully documented in the patient's record.

Regarding clinical care, one of the two points for improvement was noted for both Hospitals A and B. These hospitals reported that the highest percentage of nonsecured IDCs was mainly in ICUs with 75% of all ICU patients having a nonsecured IDC. The reason for nonsecured IDC given by nursing staff was many of the patients were sedated and thus immobile and would not be inadvertently pulling on the IDC. However, patients with IDCs in situ may experience untoward traction on the IDC when staff are dealing with equipment, bed rails and the like while giving care. The nurses' attitude around the poor rates of catheter securement demonstrates an urgent need for education especially around the potential of bladder neck trauma if not secured correctly (Macneil et al. 2018). The adverse consequences of unsecured or incorrectly secured IDC may also include pressure injury at the meatus (Munien et al. 2024). The continual friction of a nonsecured device may also contribute to an increased risk of CAUTI due to mucosal injury and microorganism infiltration (Feneley, Hopley, and Wells 2015).

In addition to securing the catheter, the date of the last change of the drainage bag was poorly documented. Standardisation of the day of the week to change catheter bags in ward areas, for example, promoting ‘Wee Wednesdays’, may be of benefit. As a further reminder, identifying the change date by either using a sticker or writing the date on the catheter drainage bag and the care plan may assist as ongoing reminders.

There was a lack of documented perianal and penile hygiene. The most common infection associated with short‐term catheterisation is the transient bacteria *Escherichia coli* which commonly resides within the gastrointestinal tract (Ramanathan and Duane 2014). Transient bacteria are those bacteria that have been temporarily displaced from their normal environment, therefore, residual occult faecal matter around the perineum is an example of transient bacteria (Jacobsen et al. 2008). The need for appropriate perineal hygiene (The Joanna Briggs Institute 2012) to minimise the risk of CAUTI should be reinforced to all staff and education around the importance of such practice in reducing CAUTI. A recent study identified that nurses were unaware that using disinfectant was unnecessary during insertion (Webster et al. 2001). This may be because nurses recognise the risk of microorganism transmission during insertion. However, there are no data suggesting they recognise the importance of perineal/penile hygiene to prevent ongoing contamination (Balu et al. 2021).

The intention, following this study, was to use the results to form recommendations to assist in reducing the incidence of CAUTIs. As a result, education in all aspects of IDC insertion and management, including perineal and penile hygiene for all patients with an IDC and the awareness of the need for catheter securement to thigh, is ongoing in the three hospitals. These aspects of care should be included within existing policy with adjunct re‐education of staff, particularly in ICU areas. For these reasons, this study urges the need to review current local policies and processes using best evidence clinical practice to prevent CAUTIs. Any changes to policy should be translated into practice facilitated by education of clinical staff and followed up to monitor staff uptake and adherence.

Any electronic documentation systems should incorporate the mandatory fields that mimic best evidence for the insertion and management of IDCs. The output details (urinary catheter) should be modified to allow the documentation of all details around IDC insertion. As this involves a software modification, we would suggest an immediate/interim solution is to provide a template placed in the paper documentation or into eMR to prompt all clinicians to insert an IDC to document important details.

The strength of this study was that it was a multisite, point prevalence study involving three hospitals. This gave us the opportunity to study a total of 1730 patients, which is a strength. In addition, a further strength was that each of these organisations had different documentation systems. Comparisons of documentation compliance allowed for loose comparisons of documentation systems. A further strength was that the data collectors worked in teams of two which helped ensure the accuracy of data collection that served a nonbiased approach.

The limitations of this study included that each member of the audit team worked within the organisation they were auditing which opened a potential bias. In an ideal world, each team would be independent of the organisation. However, due to funding issues, this was not possible.

## 5 Conclusion

This cross‐sectional design study investigated IDC use in three Australian hospitals. The overall mean point prevalence of 12.9% was within the range of published literature. Several inconsistencies across the three sites were observed. These included the varying documentation modes with varying completeness of IDC insertion documentation. Documentation was better when a template was used. Improvements across all three sites are required, specifically around securing the urinary catheter to the patient's thigh within ICU and documentation of the urine bag change in all wards. Further work is needed to ensure nurses are aware of the adverse effects of urinary catheters and adhere to best practice guidelines.

## Conflicts of Interest

The authors declare no conflicts of interest.

## Supporting information


Data S1.

## Data Availability

The data that support the findings of this study are available on request from the corresponding author. The data are not publicly available due to privacy or ethical restrictions.

## References

[jocn17459-bib-0001] Adib‐Hajbaghery, M. , and M. Aghajani . 2009. “Quality of Care for Patients With Indwelling Urinary Catheter in Selected Hospitals in Kashan, Iran 2007.” International Journal of Urological Nursing 3, no. 2: 43–49.

[jocn17459-bib-0002] Agency for Healthcare Research and Quality . 2015. “Technical Interventions to Prevent CAUTI.” Rockville, MD.

[jocn17459-bib-0003] Andreessen, L. , M. H. Wilde , and P. Herendeen . 2012. “Preventing Catheter‐Associated Urinary Tract Infections in Acute Care: The Bundle Approach.” Journal of Nursing Care Quality 27, no. 3: 209–217. 22327333 10.1097/NCQ.0b013e318248b0b1

[jocn17459-bib-0004] Appah, Y. , K. F. Hunter , and K. N. Moore . 2016. “Securement of the Indwelling Urinary Catheter: A Prevalence Study.” Journal of Wound Ostomy & Continence Nursing 43, no. 2: 173–177. 10.1097/WON.0000000000000176 26418849

[jocn17459-bib-0005] Australian Commission on Safety and Quality in Health Care . 2018. “Healthcare Associated Infections.” Accessed 3 June, 2024. www.safetyandquality.gov.au.

[jocn17459-bib-0006] Balu, P. , D. Ravikumar , V. Somasunder , et al. 2021. “Assessment of Knowledge, Attitude and Practice on Prevention of Catheter‐Associated Urinary Tract Infection (CAUTI) Amongst Health Care Professionals Working in a Tertiary Care Teaching Hospital.” Journal of Pure and Applied Microbiology 15, no. 1: 335–345.

[jocn17459-bib-0007] Coventry, L. , V. Patton , A. Whyte , et al. 2021. “Adherence to Evidence‐Based Guidelines for Indwelling Urinary Catheter Management: A Cross‐Sectional Study.” Collegian 28, no. 5: 515–520.

[jocn17459-bib-0008] Cromwell, K. , and J. Crespo‐Diaz . 2014. “Implementation and Sustainment of Hospital‐Wide Evidence Based Practice (EBP) Bundles to Prevent Catheter Associated Urinary Tract Infections (CAUTI).” American Journal of Infection Control 42, no. 6: S21–S22.

[jocn17459-bib-0009] d'Stere Murphy, B. P. , S. E. Harper , M. A. Slavin , and L. J. Worth . 2018. “Development and Utility of a Standardized Bedside Audit Tool to Monitor Indwelling Urinary Catheter Use in Patients With Cancer.” Urologic Nursing 38, no. 3: 167–172.

[jocn17459-bib-0010] Duclos‐Miller, P. 2016. Improving Nursing Documentation and Reducing Risk. Brentwood, TN, USA: HCPro.

[jocn17459-bib-0011] Emberton, M. , and J. M. Fitzpatrick . 2008. “The Reten‐World Survey of the Management of Acute Urinary Retention: Preliminary Results.” BJU International 101, no. s3: 27–32. 18307683 10.1111/j.1464-410X.2008.07491.x

[jocn17459-bib-0012] Feneley, R. , I. Hopley , and P. Wells . 2015. “Urinary Catheters: History, Current Status, Adverse Events and Research Agenda.” Journal of Medical Engineering & Technology 39, no. 8: 459–470. 26383168 10.3109/03091902.2015.1085600 PMC4673556

[jocn17459-bib-0013] Fernández‐Ruiz, M. , B. Calvo , R. Vara , R. Villar , and J. Aguado . 2013. “Inappropriate Use of Urinary Catheters in Patients Admitted to Medical Wards in a University Hospital.” Enfermedades Infecciosas y Microbiología Clínica 31, no. 8: 523–525. 23601704 10.1016/j.eimc.2013.02.013

[jocn17459-bib-0014] Fink, R. , H. Gilmartin , A. Richard , E. Capezuti , M. Boltz , and H. Wald . 2012. “Indwelling Urinary Catheter Management and Catheter‐Associated Urinary Tract Infection Prevention Practices in Nurses Improving Care for Healthsystem Elders Hospitals.” American Journal of Infection Control 40, no. 8: 715–720. 22297241 10.1016/j.ajic.2011.09.017

[jocn17459-bib-0015] Flores‐Mireles, A. , T. Hreha , and D. Hunstad . 2019. “Pathophysiology, Treatment, and Prevention of Catheter‐Associated Urinary Tract Infection.” Topics in Spinal Cord Injury Rehabilitation 25, no. 3: 228–240. 31548790 10.1310/sci2503-228 PMC6743745

[jocn17459-bib-0016] Foxman, B. 2010. “The Epidemiology of Urinary Tract Infection.” Nature Reviews. Urology 7, no. 12: 653–660. 21139641 10.1038/nrurol.2010.190

[jocn17459-bib-0017] Giles, M. , L. Graham , J. Ball , et al. 2019. “Variations in Indwelling Urinary Catheter Use in Four Australian Acute Care Hospitals.” Journal of Clinical Nursing 28: 4572–4581. 31469471 10.1111/jocn.15048

[jocn17459-bib-0018] Govasli, L. , and B. Solvoll . 2020. “Nurses' Experiences of Busyness in Their Daily Work.” Nursing Inquiry 27, no. 3: e12350. 32133740 10.1111/nin.12350

[jocn17459-bib-0019] Gustafsson, U. , M. Scott , M. Hubner , et al. 2019. “Guidelines for Perioperative Care in Elective Colorectal Surgery: Enhanced Recovery After Surgery (ERAS®) Society Recommendations: 2018.” World Journal of Surgery 43, no. 3: 659–695. 30426190 10.1007/s00268-018-4844-y

[jocn17459-bib-0020] Hernandez, M. , A. King , and L. Stewart . 2019. “Catheter‐Associated Urinary Tract Infection (CAUTI) Prevention and Nurses' Checklist Documentation of Their Indwelling Catheter Management Practices.” Nursing Praxis in New Zealand 35, no. 1: 29–42.

[jocn17459-bib-0021] Jacobsen, S. , D. Stickler , H. Mobley , and M. Shirtliff . 2008. “Complicated Catheter‐Associated Urinary Tract Infections due to *Escherichia coli* and *Proteus mirabilis* .” Clinical Microbiology Reviews 21, no. 1: 26–59. 18202436 10.1128/CMR.00019-07 PMC2223845

[jocn17459-bib-0022] Jones, A. , C. Nagle , T. Ahern , and W. Smyth . 2022. “Evidence for a Nurse‐Led Protocol for Removing Urinary Catheters: A Scoping Review.” Collegian Journal of the Royal College of Nursing Australia 30, no. 1: 190–197.

[jocn17459-bib-0023] Kelleher, M. M. 2002. “Removal of Urinary Catheters: Midnight vs 0600 Hours.” British Journal of Nursing 11, no. 2: 84–90. 11823735 10.12968/bjon.2002.11.2.9308

[jocn17459-bib-0024] Leranoz, S. , M. Orus , F. Berlanga , F. Dalet , and M. Vinasm . 1997. “New Fimbrial Adhesins of Serratia Marcescens Isolated From Urinary Tract Infections: Description and Properties.” Journal of Urology 157: 694–698. 8996400

[jocn17459-bib-0025] Macneil, J. , R. Wilkins , R. Taylor , and H. Lau . 2018. “Does the Method of “Securing the Catheter” Make Any Difference?” Journal of Clinical Urology 11, no. 1: 21–26.

[jocn17459-bib-0026] McCarthy, B. , S. Fitzgerald , M. O'Shea , et al. 2019. “Electronic Nursing Documentation Interventions to Promote or Improve Patient Safety and Quality Care: A Systematic Review.” Journal of Nursing Management 27, no. 3: 491–501. 30387215 10.1111/jonm.12727

[jocn17459-bib-0027] Meillat, H. , C. Magallon , C. Brun , et al. 2021. “Systematic Early Urinary Catheter Removal Integrated in the Full Enhanced Recovery After Surgery (ERAS) Protocol After Laparoscopic Mid to Lower Rectal Cancer Excision: A Feasibility Study.” Annals of Coloproctology 37, no. 4: 204–211. 33887815 10.3393/ac.2020.05.22 PMC8391039

[jocn17459-bib-0028] Munien, K. , K. Ravichandran , H. Flynn , et al. 2024. “Catheter‐Associated Meatal Pressure Injuries (CAMPI) in Patients With Long‐Term Urethral Catheters‐A Cross‐Sectional Study of 200 Patients.” Translational Andrology and Urology 13, no. 1: 42–52. 38404556 10.21037/tau-23-445 PMC10891379

[jocn17459-bib-0029] Mutshatshi, T. , T. Mothiba , P. Mamogobo , and M. Mbombi . 2018. “Record‐Keeping: Challenges Experienced by Nurses in Selected Public Hospitals.” Curationis 41, no. 11: e1–e6. 10.4102/curationis.v41i1.1931 PMC6111626 30198294

[jocn17459-bib-0030] Okeke, C. , M. Ogunjimi , E. Jeje , A. Obi , and C. Uzoma . 2023. “Urinary Catheter Documentation in a Nigerian Teaching Hospital: Are We Recording Enough?” Journal of the West African College of Surgeons 13, no. 2: 45–48. 37228889 10.4103/jwas.jwas_288_22 PMC10204908

[jocn17459-bib-0031] Pandian, S. , and M. Drake . 2016. “Retention of Urine.” In WOCN Core Curriculum—Continence Management. Philadelphia, edited by D. B. Doughty and K. N. Moore , 72–91. Philadelphia, USA: Wolters Kluwer Health.

[jocn17459-bib-0032] Parker, V. , M. Giles , L. Graham , et al. 2017. “Avoiding Inappropriate Urinary Catheter Use and Catheter‐Associated Urinary Tract Infection (CAUTI): A Pre‐Post Control Intervention Study.” BMC Health Services Research 17: 1–9. 28464815 10.1186/s12913-017-2268-2 PMC5414128

[jocn17459-bib-0033] Ramanathan, R. , and T. Duane . 2014. “Urinary Tract Infections in Surgical Patients.” Surgical Clinics of North America 94, no. 6: 1351–1368. 25440128 10.1016/j.suc.2014.08.007

[jocn17459-bib-0034] Rhodes, D. , J. Kennon , S. Aitchison , et al. 2014. “Improvements in Process With a Multimodal Campaign to Reduce Urinary Tract Infections in Hospitalised Australian Patients.” Healthcare Infection 19, no. 4: 117–121.

[jocn17459-bib-0035] Shackley, D. , C. Whytock , G. Parry , et al. 2017. “Variation in the Prevalence of Urinary Catheters: A Profile of National Health Service Patients in England.” BMJ Open 7, no. 6: e013842. 10.1136/bmjopen-2016-013842 PMC5577876 28645950

[jocn17459-bib-0036] Shamshiri, M. , B. Suh , N. Mohammadi , and R. Amjad . 2016. “A Survey of Adherence to Guidelines to Prevent Healthcare‐Associated Infections in Iranian Intensive Care Units.” Iranian Red Crescent Medical Journal 18, no. 6: 1–8. 10.5812/ircmj.27435 PMC5004621 27621932

[jocn17459-bib-0037] So, K. , D. Habashy , B. Doyle , and L. Chan . 2014. “Indwelling Urinary Catheters: Pattern of Use in a Public Tertiary‐Level Australian Hospital.” Urologic Nursing 34, no. 2: 69–73. 24919244

[jocn17459-bib-0038] The Joanna Briggs Institute . 2012. Management of Short‐Term Indwelling Urethral Catheters to Prevent Urinary Tract Infections. 2008‐2011: Evidence‐Based Information Sheets for Health Professionals: Best Practice, 119–122. South Australia: Wiley‐Blackwell.

[jocn17459-bib-0039] von Elm, E. , D. Altman , M. Egger , S. Pocock , P. Gøtzsche , and J. Vandenbroucke . 2008. “STROBE Initiative. The Strengthening the Reporting of Observational Studies in Epidemiology (STROBE)statement: Guidelines for Reporting Observational Studies.” Journal of Clinical Epidemiology 61, no. 4: 344–349. 18313558 10.1016/j.jclinepi.2007.11.008

[jocn17459-bib-0040] Warren, J. 2001. “Catheter‐Associated Urinary Tract Infections.” International Journal of Antimicrobial Agents 17: 299–303. 11295412 10.1016/s0924-8579(00)00359-9

[jocn17459-bib-0041] Webster, J. , R. Hood , C. Burridge , M. Doidge , K. Phillips , and N. George . 2001. “Water or Antiseptic for Periurethral Cleaning Before Urinary Catheterization: A Randomized Controlled Trial.” American Journal of Infection Control 29, no. 6: 389–394. 11743486 10.1067/mic.2001.117447

[jocn17459-bib-0042] Werneburg, G. 2022. “Catheter‐Associated Urinary Tract Infections: Current Challenges and Future Prospects.” Research and Reports in Urology 4, no. 14: 109–133. 10.2147/RRU.S273663 PMC8992741 35402319

[jocn17459-bib-0043] Wynne, R. , M. Patel , N. Pascual , et al. 2013. “A Single Centre Point Prevalence Survey to Determine Prevalence of Indwelling Urinary Catheter Use and Nurse‐Sensitive Indicators for the Prevention of Infection.” Healthcare Infection 19, no. 1: 13–19.

